# The Claudin-Low Subtype of High-Grade Serous Ovarian Carcinoma Exhibits Stem Cell Features

**DOI:** 10.3390/cancers13040906

**Published:** 2021-02-22

**Authors:** Chiara Romani, Davide Capoferri, Elisabetta Grillo, Marco Silvestri, Michela Corsini, Laura Zanotti, Paola Todeschini, Antonella Ravaggi, Eliana Bignotti, Franco Odicino, Enrico Sartori, Stefano Calza, Stefania Mitola

**Affiliations:** 1 Angelo Nocivelli Institute of Molecular Medicine, University of Brescia and ASST Spedali Civili di Brescia, 25123 Brescia, Italy; davide.capoferri@unibs.it (D.C.); laura.zanotti84@gmail.com (L.Z.); todeschini.paola@gmail.com (P.T.); antonella.ravaggi@unibs.it (A.R.); bignottieliana@gmail.com (E.B.); 2 Department of Molecular and Translational Medicine, University of Brescia, 25123 Brescia, Italy; elisabetta.grillo@unibs.it (E.G.); marcosilvestri.1992@gmail.com (M.S.); michela.corsini@unibs.it (M.C.); 3 Biomarkers Unit, Department of Applied Research and Technological Development, Fondazione IRCCS Istituto Nazionale dei Tumori di Milano, 20133 Milano, Italy; 4 Division of Obstetrics and Gynecology, ASST Spedali Civili di Brescia, 25123 Brescia, Italy; franco.odicino@unibs.it (F.O.); enrico.sartori@unibs.it (E.S.); 5 Department of Clinical and Experimental Sciences, Division of Obstetrics and Gynecology University of Brescia, 25123 Brescia, Italy; 6 BDbiomed, Big & Open Data Innovation Laboratory, University of Brescia, 25123 Brescia, Italy

**Keywords:** serous ovarian cancer, molecular profiling, claudin-low, cancer stem cells, EMT

## Abstract

**Simple Summary:**

Here, we identified and characterized a claudin-low subtype of high-grade serous ovarian cancer. This rare variant of undifferentiated neoplasm shares transcriptional features with the homonym subtype of breast cancer, including low epithelial differentiation, high mesenchymal signature, and enrichment for stem cell features. Since the claudin-low transcriptional signature is associated with poor prognosis, we believe that the identification of the claudin-low molecular profile may have important clinical implications, paving the way for personalized medicine in ovarian cancer patients.

**Abstract:**

Claudin-low cancer (CL) represents a rare and biologically aggressive variant of epithelial tumor. Here, we identified a claudin-low molecular profile of ovarian high-grade serous carcinoma (HGSOC), which exhibits the main characteristics of the homonym breast cancer subtype, including low epithelial differentiation and high mesenchymal signature. Hierarchical clustering and a centroid based algorithm applied to cell line collection expression dataset labeled 6 HGSOC cell lines as CL. These have a high energy metabolism and are enriched in CD44^+^/CD24^−^ mesenchymal stem-like cells expressing low levels of cell-cell adhesion molecules (claudins and E-Cadherin) and high levels of epithelial-to-mesenchymal transition (EMT) induction transcription factors (Zeb1, Snai2, Twist1 and Twist2). Accordingly, the centroid base algorithm applied to large retrospective collections of primary HGSOC samples reveals a tumor subgroup with transcriptional features consistent with the CL profile, and reaffirms EMT as the dominant biological pathway functioning in CL-HGSOC. HGSOC patients carrying CL profiles have a worse overall survival when compared to others, likely to be attributed to its undifferentiated/stem component. These observations highlight the lack of a molecular diagnostic in the management of HGSOC and suggest a potential prognostic utility of this molecular subtyping.

## 1. Introduction

High-grade serous carcinoma (HGSOC) is the most frequent and aggressive histological type of epithelial ovarian cancer, and the main cause of mortality for gynecological neoplasms. Despite cytoreductive surgery followed by platinum-based adjuvant chemotherapy has made some progress in improving the survival rate of patients with HGSOC, the vast majority of them experience recurrence and platinum resistance. For these patients, the 5-year survival rate dramatically falls below 30% [1,2]. The main reasons for this poor prognosis is attributable to the fact that the vast majority of these patients are diagnosed at advanced disease due to the lack of effective clinical markers and of an identifiable precursor lesion [3]. Moreover, the clinical evolution of the disease depends on tumor biology that remains largely unknown. This also reduces the efficacy of treatment with the conventional chemotherapy.

Several genomic predictors have been designed to stratify HGSOC into prognostic subgroups, in an attempt to categorize patients based on a specific molecular signature that could predict survival, relapse, response to chemotherapy [4,5,6], as well as residual disease after primary debulking surgery, potentially acting as a biomarker that guides surgical decisions [7]. However, their application in clinical settings remains limited [8,9], in part because of the high heterogeneity of the disease, which makes it difficult to mutually assign a tumor sample to a single molecular subtype [10].

In breast cancer (BC), the intrinsic molecular subtypes originally identified by gene expression profiling [11,12,13,14] and further confirmed by immunohistochemical evaluation of hormone receptors and human epithelial growth factor 2 (HER2) gene amplification [15] have quickly entered clinical practice, complementing and expanding the information provided by classical pathological markers. This sub-classification is currently used to stratify patients for prognosis prediction, and to select treatments. The basal-like subtype of breast cancer, commonly known as triple-negative (TNBC) since the majority of cases lack expression of estrogen and progesterone receptors and are devoid of overexpression and/or amplification of HER2, has been associated with HGSOC by spectrum and frequency of genomic mutations. Specifically, analogies regard p53 and BRCA genes, suggesting similar initiating genetic events that drive both basal-like and serous ovarian carcinogenesis [16]. Among TNBC, the most recently identified subtype is the claudin-low (CL). CL tumors account for 7 to 14% of all invasive breast cancer [17] and are characterized by the loss of genes involved in cell-cell adhesion, the enrichment of epithelial-to-mesenchymal (EMT) transcription markers and the stem cell-like features. Clinically, CL breast cancers have high rates of metastases and recurrence, and are associated with poor prognosis [18,19,20,21].

More recently, a CL subtype of high-grade, muscle-invasive urothelial carcinoma (UC) has been described and it is characterized by similar biologic features of the homonym subtype of breast cancer [22].

To date a consensus molecular based classification of HGSOC is still not well defined. Here we reported the identification of a novel molecular subtype of HGSOC exhibiting all the characteristic features of CL tumors, including low expression of tight and adherent junctions, EMT markers and stem cell like characteristics. We used the transcriptional signature of CL tumors to distinguish CL from other HGSOC cell lines. The CL-HGSOC cell lines maintained in vitro all the features of CL tumors, including the expression of EMT markers and the stem cell characteristics. Finally, analyzing the multiple publicly available microarray datasets of primary HGSOC, we identified a highly homogeneous group of CL tumors. Of note, patients harboring CL tumors showed significantly reduced survival.

## 2. Materials and Methods

### 2.1. Data Sources

Breast and ovarian cancer cell lines public microarray datasets were obtained from The Cancer Cell Line Encyclopedia (CCLE) database (https://portals.broadinstitute.org/ccle, accessed on 1 February 2019) [23].

Ovarian cancer gene expression data were downloaded from the Curated Ovarian Data [24]. All tumors annotated as high-grade serous ovarian cancers and with large sample-size (*n* > 100) microarray data sets were included in the analysis, including the following: TCGA [25], the Bonome’s dataset (GSE26712) [26], the Tothill’s dataset (GSE9891) [27], the Japanese dataset (GSE32062-GPL6480) [28]. Clinical information on stage, grade and survival time was obtained from the corresponding article and website.

### 2.2. Cell Culture

Ovarian cancer cell lines OVCAR3 and OV7 were purchased respectively from the European Collection of Authenticated Cell Cultures (ECACC) and Istituto Zooprofilattico Sperimentale della Lombardia e dell’Emilia (IZS, Brescia, Italy), and cultured using medium and supplements recommended by the supplier.

### 2.3. Chemoresistance Assay

The 6 × 10^3^ cells/cm^2^ were cultured in growth medium in the absence or the presence of carboplatin (from 9.4 to 300 µM). After 48 h cells number was evaluated by crystal violet colorimetric assay (OD at 595 nm) [29].

### 2.4. Soft Agar Assay

Cells (5 × 10^4^) were suspended in 3 mL of complete growth medium containing 0.3% agar and poured on to 2 mL pre-solidified 0.6% agar in a 6-well plate. After 3 weeks of incubation, colonies were observed under a phase contrast microscope, photographed, and their area was measured using the Image J Software and the SA_NJ algorithm [30].

### 2.5. Quantitative PCR

cDNA was generated from total RNA using the SuperScriptII reverse transcriptase (Invitrogen). RT-qPCR for targets (Table 1) and HPRT1 and PPIA reference genes was established in a multiplex procedure for simultaneous amplification of each template, as described by our group [31].

### 2.6. Western Blot Analysis

Cells were grown up to 70% confluence. Whole-cell lysates were prepared in RIPA lysis buffer (Thermo Fisher Scientific, Waltham, MA, USA) with protease inhibitors. Proteins were separated by SDS-PAGE and probed with specific antibodies and HRP-labeled secondary antibody (Santa Cruz Biotech, Heidelberg, Germany). Chemiluminescent signal was acquired by ChemiDoc Imaging System (BioRad, Hercules, CA, USA). The original western blotting figures can be found in Figure S4.

### 2.7. Flow Cytometry and Cell Sorting

OVCAR3 and OV7 cells were harvested using Trypsin-EDTA solution and stained with PE-Cy5-anti-CD117 (YB5.B8, BD), PE-Cy7-anti-CD24 (M1/69, Thermo Fisher) and APC-anti-CD44 (IM7, Thermo Fisher). Aldehyde dehydrogenase 1 (ALDH-1) activity was detected by ALDEFLUOR Assay (STEM CELL Technologies, Vancouver, BC, Canada) according to the manufacturer’s instructions.

Cells were analyzed by BD FACSCanto II (Becton Dickinson, Franklin Lakes, NJ, USA) and FACS DIVA software. Samples were compensated using UltraComp eBeads (InvitroGen, Waltham, MA, USA). Duplets and dead cells were removed from analysis by Violet Dead Cell Stain Kit 405 nm (Thermo Fisher). Specific labelling was controlled by Fluorescence Minus One (FMO) stained cells. CD44^+^CD24^−^ cells were sorted by BD FACSAria (Becton Dickinson, Milano, Italy).

### 2.8. Metabolic Analysis

Oxygen consumption rate (OCR) and Extracellular acidification rate (ECAR) measurements were performed at 6 min intervals (2 min mixing, 2 min waiting and 2 min measuring) using a Seahorse XFe24 Extracellular Flux Analyzer (XFe Wave software) (Agilent, Santa Clara, CA, USA). Cells were seeded on Seahorse XFe24 culture plates (Agilent) previously treated with 3.5 mg/cm^2^ Corning Cell-Tak Cell and Tissue Adhesive (see [32]). Seahorse XF Mito-Stress Test was used to measure key parameters of mitochondrial function. Sequential treatments with Oligomycin (1 μM final), FCCP (0.5 μM final), and Rotenone/Antimycin A (0.5 μM final) were performed to enable quantification of basal OCR, ATP-coupled OCR, proton leak, and maximal respiration.

### 2.9. Data processing and Statistical Analysis

Expression data and clinical information about FIGO stage, tumor grade and patient survival were obtained from curatedOvarianData R package [24]. Gene expression values were log2 transformed and normalized using quantile normalization. An unsupervised hierarchical clustering analysis with Ward agglomeration method [33] and Euclidean distance metric was used to identify the molecular subtypes.

Gene level linear models with moderated *t*-test statistics [34] were performed to identify differentially expressed genes between claudin-low and other HGSOC samples. The threshold values for DEG were a log2 fold-change (FC) in expression >2 and *p* < 0.01. Gene Set Enrichment analysis was performed using GSEA (Hallmark gene set collection) on the gene list ranked according to a moderated *t*-statistics, comparing patients with CL and non-CL profile.

A CL centroid-based predictor was built following Prat et al. [19]. A centroid was defined as the average expression vector of selected genes separately for breast cancer (BC) cell-lines in the CL (*n* = 9) or other groups (*n* = 43), as defined by Prat et al., using data available in the *genefu* package [35]. Each cell line was assigned either to CL or “other” according to which centroid (either “claudin-low” or “others”) the cell-line is closest to in the Euclidean space defined by the gene list.

All analyses were performed using R (version 4.03) [36]. 

## 3. Results

### 3.1. Identification of Claudin-Low Ovarian Cancer Cell Lines

**Figure 1:**
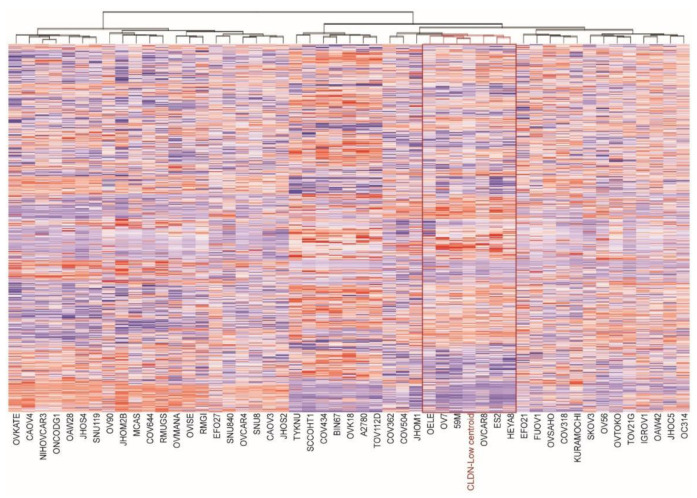
Identification of the claudin-low subtype in CCLE ovarian cancer cell lines. Hierarchical clustering using Euclidean distance metric and Ward algorithm was computed to graphically show the similarity between ovarian cell lines and claudin low (CLDN-low) centroid used for their classification. In the tree, the red node indicates the most highly correlated cell lines displaying similar gene expression patterns as breast claudin-low subtype.

We first applied the claudin-low (CL) centroid predictor from Prat original data to BC cell lines included in the CCLE collection using 775 genes out of the 807 originally proposed (32 were not present in the CCLE data). The centroid predictor assigned the CL label to BT549, HS578T, MDA-MB-157, MDA-MB-231, MDA-MB-436, CAL120, HCC1395 and HS274T cell lines, while the remaining 44 BC cell lines were labelled as “others” (from here on, non-claudin-low cancer will be defined as “other”). In order to explore the existence of a CL subtype within HGSOC cell lines, we applied the centroid-predictor used for BC cell lines to the ovarian cancer cell lines. Among them, OELE, OV7, 59M, OVCAR8, ES2 and HEYA8 cell lines were labeled as CL cells by the centroid predictor (Figure 1). GSEA performed on genes ranked according to the *t*-statistic comparing CL versus “others”, revealed a strong and significant positive enrichment for EMT, cell cycle, proliferation and hypoxia gene signatures (FDR < 0.001, Figure S1).

**Figure 2:**
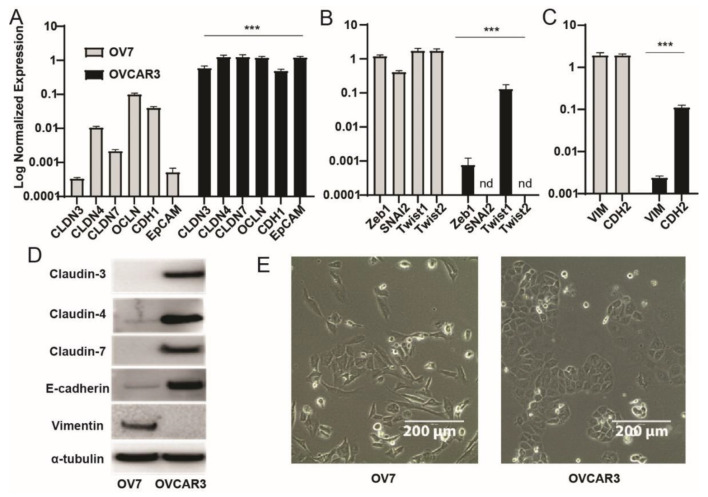
OV7 cells express EMT and stem cell markers. (**A**–**C**) RT-qPCR measurement of mRNA levels of epithelial markers, EMT-regulating transcription factors and mesenchymal markers in OV7 and OVCAR3 cells. Values are given as normalized expression relative quantification and displayed on Log scale. Error bars represent the mean ± SD of three independent experiments. ***, *p* < 0.001 independent samples *t*-test comparing individual markers between OV7 and OVCAR3 cells. (**D**) OV7 and OVCAR3 cell lysates were analyzed by western blot analysis with epithelial-specific (rabbit anti-claudin 3, mouse anti-claudin 4, mouse anti-claudin 7, ThermoFisher Scientific, mouse anti-E-cadherin, R&D System) and mesenchymal-specific antibodies (mouse anti-Vimentin, Sigma-Aldrich) that confirmed a significant unbalance of OV7 towards a mesenchymal phenotype and of OVCAR3 towards the epithelial phenotype. Mouse anti-α-tubulin loading control (Sigma-Aldrich). (**E**) Analysis by phase-contrast microscopy showing marked morphological difference between OV7 and OVCAR3 cells (scale bar 200 µm).

To obtain information on the specific properties that differentiate CL from non-claudin-low ovarian cancer cell lines, we analyzed OV7 and OVCAR3 cell lines respectively as a model of CL and non-claudin low cell lines. First, we validated the expression of a subset of genes, including EMT and stem cell markers. As anticipated, OV7 cells show low to undetectable mRNA levels of genes encoding for the epithelial junctional complex claudin-3, -4, -7, occludin, E-Cadherin, and of the epithelial cell adhesion molecules (Ep-CAM). In addition, compared to OVCAR3, OV7 cells express higher levels of mesenchymal markers including Vimentin, N-Cadherin, EMT-inducing transcriptional factors Zeb1, Snai2, Twist1 and Twist2 (Figure 2A–C). Similar results were obtained in the well-characterized CL breast cancer cell line MDA-MB-231 [19,37] (Figure S2).

Western blot confirmed the low expression of claudins and E-cadherin with concomitant high expression of vimentin in OV7 cells and an opposite profile in OVCAR3 cells (*p* < 0.001 for all comparisons, Figure 2D and Figure S3). The two cell lines are also morphologically different, with OV7 cells displaying a spindle-like shape, typically acquired during the EMT process, while OVCAR3 cells maintain the epithelial-like shape (Figure 2E).

### 3.2. Ovarian Claudin-Low Cells Display Stem Cell Characteristics

**Figure 3:**
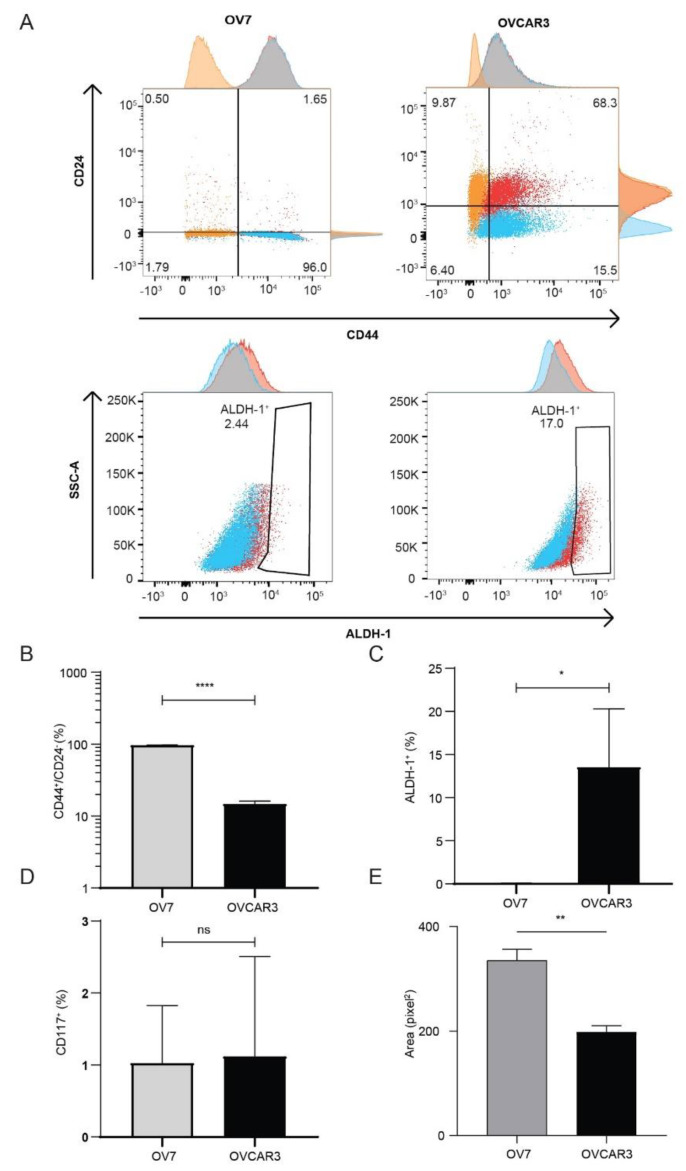
OV7 cells are enriched in CD24^−^/CD44^+^ cells. (**A**–**C**) OV7 and OVCAR3 cells were immunostained with CD44, CD24, ALDEFLUOR or (**D**) CD117, and analyzed by FACS. (**E**) OV7 and OVCAR3 cells were embedded in soft agar to measure anchorage-independent cell growth. Bars represent the mean ± SD of three independent experiments. ****, *p* < 0.0001, **, *p* < 0.001, *, *p* < 0.01 Mann-Whitney test.

Previous studies have linked CL breast tumor with stem cell-like features, inferring that this tumor subtype might be enriched for cancer initiating cells and arises from malignant transformation of mammary stem cells [38]. Indeed, the loss of the epithelial architecture observed in OV7, along with the acquisition of mesenchymal features throughout the EMT process, may reflect the arrest of these cells in an early stage of stem-to-epithelial differentiation. Since in breast and ovary cancers, two main populations of cancer stem cells (CSCs) have been characterized according to the expression of CD44^+^/CD24^−^ (mesenchymal-like CSC) or ALDH+ (epithelial-like CSC) [39,40,41], both endowed with tumor-initiating potential, we tested OV7 and OVCAR3 for expression of CD117 (c-kit), CD44, CD24 and for the enzymatic activity of aldehyde dehydrogenase 1 (ALDH1) (Figure 3A–D). Surprisingly, only about 1% of both cell lines express CD117, the tyrosine kinase receptor associated with cancer progression and normal stem cell maintenance (Figure 3D).

Regarding the expression of CD44 and CD24, 96.63% of OV7 cells and only 14.73% of OVCAR3 are CD44^+^/CD24^−^ (Figure 3A,B), while ALDH1 activity is significantly higher in OVCAR3 (13.52%) than in OV7 cells (0.03%) (Independent samples *t*-test, *p* < 0.001, Figure 3A–C). These results suggest that, similarly to the CL subtype of breast cancer, also HGSOC cell lines contain a subpopulation of CD44^+^/CD24^−^ mesenchymal-like cancer stem cells with highly invasive capacity. In accordance with the CD44/CD24 expression profile, OV7 cells display a higher clonogenic capacity than OVCAR3 cells when cultured in anchorage-independent conditions (Figure 3E).

**Figure 4:**
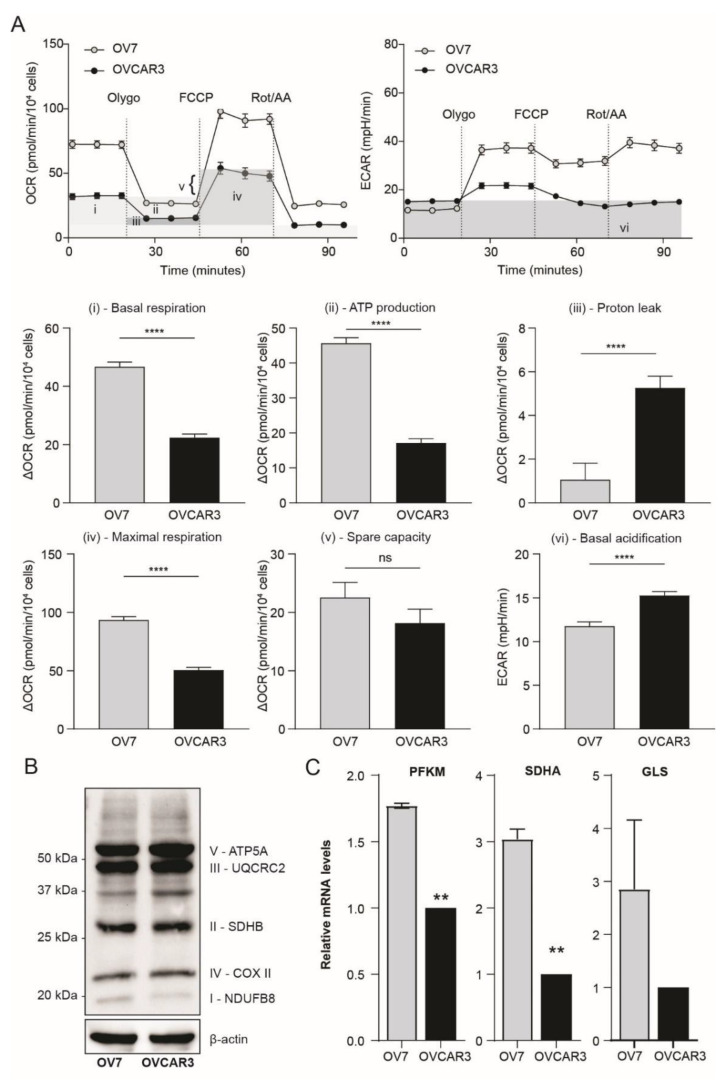
CL HGSOC cells have a high energy metabolism. (**A**) Seahorse XF Cell Mito Stress Test (OCR: oxygen consumption rate; ECAR: extracellular acidification rate) performed by Seahorse platform in OV7 and OVCAR3 cells. Sequential treatments with Oligomycin, FCCP and Rotenone/Antimycin A were performed to enable quantification of basal respiration, ATP-linked respiration, proton leak, maximal respiration, spare respiration and basal acidification. ****, *p* < 0.0001 independent sample *t* test (**B**) Western blot analysis of oxphos protein complex subunits in OV7 and OVCAR3 cells. Beta actin was used as loading control. (**C**) mRNA levels of metabolic enzymes in OV7 and OVCAR3 cells measured by RT-qPCR. **, *p* < 0.01 independent sample *t* test.

Stemness and energy metabolism are intimately entwined, thus we characterized the functional metabolic features of the CL-HGSOC cell line. Seahorse platform demonstrated that CL OV7 cells exhibit a higher energy metabolism compared to OVCAR3 cells. Oxidative phosphorylation (oxphos) measured as oxygen consumption rate (OCR) is increased with augmented basal, ATP-linked, maximal and spare respiration. On the other hand, CL cells display higher glycolytic activity, measured as extracellular acidification rate (ECAR), that is evident when mitochondrial ATP synthesis is inhibited upon oligomycin administration (Figure 4A). As shown in Figure 4B, the higher oxphos occurs in OV7 cells despite the slightly decreased levels of electron transport chain protein complexes. High levels of oxidative phosphorylation (oxphos) contribute to the maintenance of cancer stemness [42]; therefore, metabolic data further support the idea that CL HGSOC cells display stem cell features with a distinct high-oxphos metabolic profile. Energy metabolism rewiring in the OV7 cell line is paralleled by higher expression of phosphofructokinase muscle type (PFKM), glutaminase (GLS) and succinate dehydrogenase A (SDHA) metabolic enzymes (Figure 4C).

### 3.3. The CD44^+^/CD24^−^ Subpopulation of OVCAR3 Cells Resembles CL OV7 Cells

**Figure 5:**
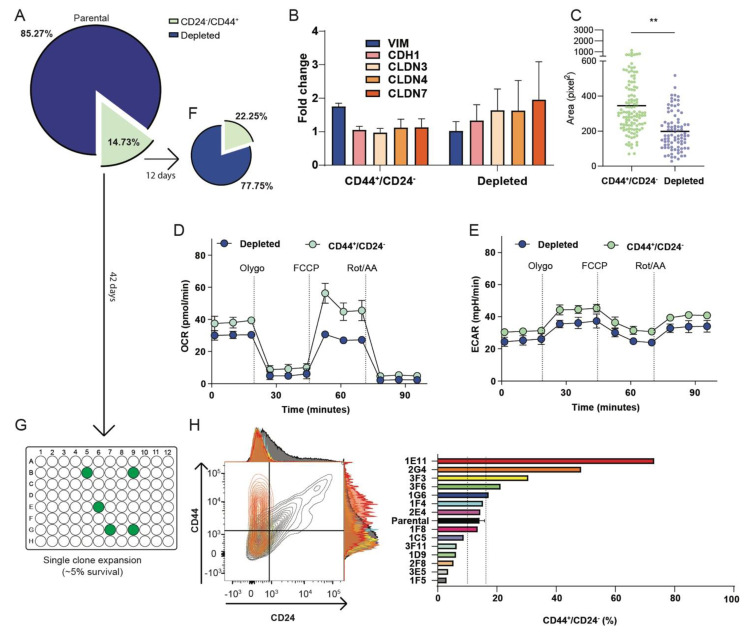
Characterization of CD44^+^/CD24^−^ subpopulation. The OVCAR3 subpopulation defined by CD44^+^/CD24^−^ phenotype was separated by cell sorting (**A**) and plated in culture for 12 days. Immunostaining with anti-CD44 and anti-CD24 followed by FACS analysis was then repeated (**F**). (**B**) Total RNA was extracted from the sorted CD44^+^/CD24^−^ cells and depleted OVCAR3, and the expression level of epithelial and mesenchymal markers was measured by RT-qPCR; bars represent mean ± SD of two independent experiments. (**C**) CD44^+^/CD24^−^ and unsorted OVCAR3 cells were embedded in soft agar to measure the anchorage-independent cell growth. **, *p* < 0.01 independent sample t test. (**D**–**E**) Energy metabolism. Seahorse XF Cell Mito Stress Test (OCR: oxygen consumption rate; ECAR: extracellular acidification rate) performed by Seahorse platform in CD44^+^/CD24^−^ and unsorted OVCAR3 cells. (**G**–**H**) The sorted CD44^+^/CD24^−^ cells as described in (**A**) were individually plated and emerging single clones analyzed by FACS for CD44 and CD24 expression.

To better characterize the identified stem-like subpopulation, we isolated CD44^+^/CD24^−^ cells from parental OVCAR3 by cell sorting and cultured for 12 days. Cytofluorimetric analysis demonstrated that after 12 days CD44^+^/CD24^−^ cells differentiated, recapitulating the features of parental (indicated as CD44^+^/CD24^−^ depleted cells) OVCAR3 cell culture (Figure 5A–E). Also, CD44^+^/CD24^−^ cells express higher levels of vimentin and lower level of epithelial markers E-cadherin and claudins compared to CD44^+^/CD24^−^ depleted cells as assessed by qPCR (Figure 5B and Figure S4). Remarkably, sorted CD44^+^/CD24^−^ OVCAR3 cells display increased energy metabolism and clonogenic capacity, similarly to CL OV7 cells (Figure 5C–E).

In parallel, sorted CD44^+^/CD24^−^ cells were individually plated to obtain single clones. After 42 days in culture, 14 grown clones were expanded and tested for expression of CD44 and CD24 (Figure 5F). Eleven out of 14 clones (78%) express CD44^+^/CD24^−^ at levels comparable to the parental or less, only 2 clones (14%) maintain the features of CL sorted cells (Figure 5G). These percentages reflect the percentage of CD44^+^/CD24^−^ cells observed in parental OVCAR3.

**Figure 6:**
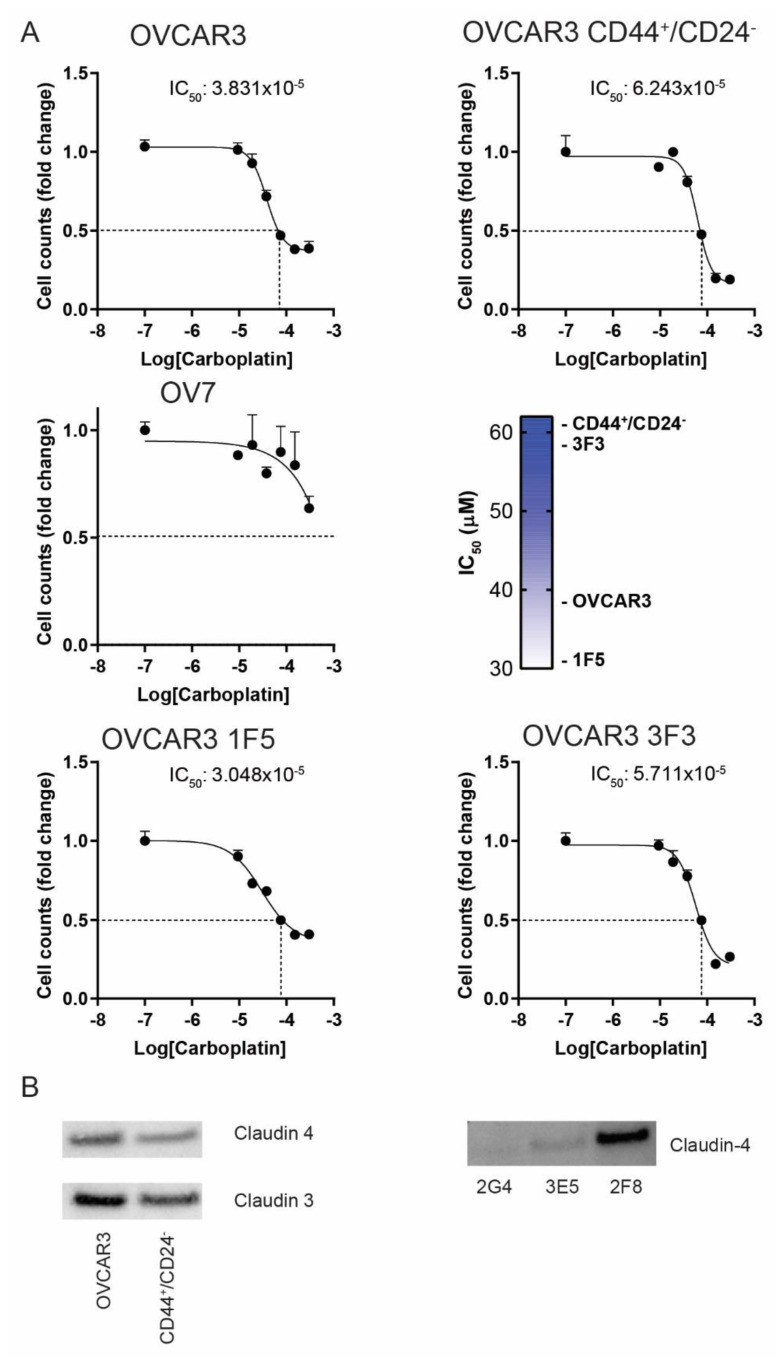
CL HGSOC cells exhibit increased resistance to carboplatin. (**A**) OV7, unsorted OVCAR3 and CD44^+^/CD24^−^ sorted OVCAR3 cell lines as well as 1F5 and 3F3 cell clones were treated with increasing doses of carboplatin (0–300 µM). After 48 h the cell number was evaluated and fold changes versus untreated cells were calculated. IC_50_ values are displayed. Values represent the mean ± SEM of two independent experiments. (**B**) Western blot analysis of Claudin expression in OVCAR3, CD44^+^/CD24^−^ sorted OVCAR3 and selected clones.

Then we assessed whether the CL cancer subtype was associated with an altered response to chemotherapy treatment. Thus, cells were exposed for 48 h to increasing doses of carboplatin. Treatment with carboplatin significantly reduces tumor cell viability. We observed that the concentration of drugs that inhibited cell viability by 50% (the IC50) was lower in OVCAR3 and 1F5 clone (38.3 and 30.4 µM) than in CD44+/CD24^−^ cells and 3F3 clone (62.4 and 57.1 µM). In that culture condition OV7 cell viability was less affected by carboplatin treatment (Figure 6), confirming that chemoresistance correlates with CD44/CD24 expression.

These data suggest that in OVCAR3, claudin loss overlaps the CD44^+^/CD24^−^ population, likely contributing to defining a mesenchymal phenotype as observed in OV7 CL cells.

### 3.4. Identification of CL Molecular Profile in HGSOC Patient Samples

**Figure 7:**
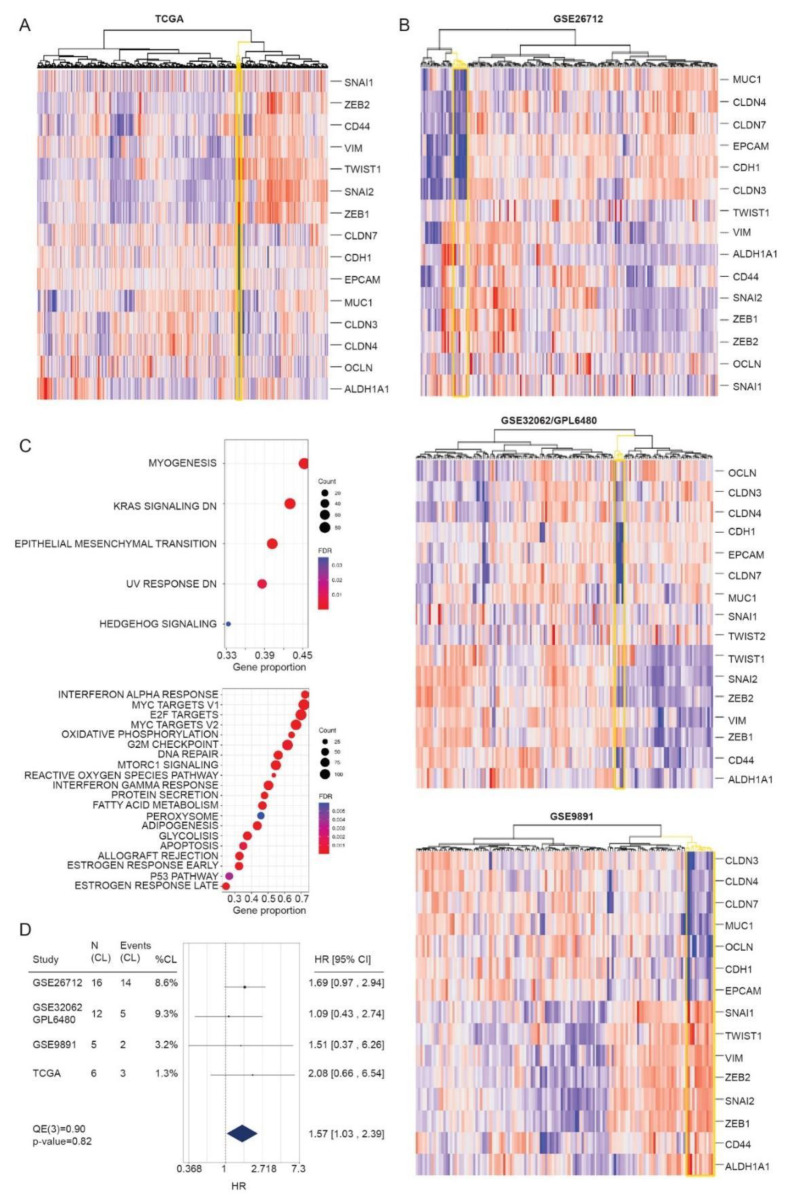
CL signature has prognostic value across multiple independent datasets. (**A**) Heat maps of gene expression values for CL signature in the TCGA cohort and (**B**) in independent datasets. A cluster emerged with transcriptomic characteristics of CL tumors (highlighted in the yellow node). (**C**) GSEA enrichment analysis for Hallmark genesets. Dot plot shows the up-regulated (upper panel) and down-regulated (lower panel) biological processes in CL-HGSOC tumors. The size of the dots represent the number of genes in the gene set, and the color of the dots represent the FDR. (**D**) Forest plot showing the meta-analysis of hazard ratios (HR) estimates for overall survival in HGSOC patients carrying CL profile. Black boxes indicate HR of individual studies.

According to the literature, the unique transcriptional features of CL tumors can be assigned to 17 key genes that strongly define this tumor subtype (Table 2). We used this minimum gene list (henceforth CL-signature) to attribute the CL profile to ovarian cancer patients, performing unsupervised hierarchical clustering on 480 advanced stage HGSOC samples from the TCGA dataset. We identified a small distinct cluster (*n* = 7, 1.5%, Figure 7A) characterized by extremely low expression of claudin 3, 4, 7, occludin, CDH1, and elevate expression of Zeb1, Snai2, Twist1 known to be transcriptional repressors of epithelial markers during EMT process. Compared to others, patients who fall into the CL group show the lowest expression of Ep-CAM, whose expression not only facilitates intercellular adhesion to ensure tissue integrity, but normally act as negative regulator of cell motility and epithelial cell migration. In fact, loss of Ep-CAM is common in tumor cells undergoing EMT, the trans-differentiation process through which epithelial cells lose their morphologic and molecular identity and adopt mesenchymal properties by virtue of actin cytoskeletal rearrangement. The expression of the ovarian carcinoma marker MUC1 is also decreased and suggests a more undifferentiated phenotype. The overall transcriptional pattern of these patients is consistent with the main features of CL tumors.

To verify the reproducibility of the analysis, we applied the CL-signature to several published HGSOC databases and platforms. Only large-size datasets of ovarian cancer annotated as serous high-grade, late stage, resulting in three microarray studies, namely GSE9891, GSE26712 and GSE32062 being included in the analysis providing data for 470 patients. In all independent cohorts, hierarchical clustering of gene expression values, using the CL minimum gene list, revealed a tumor cluster with transcriptional characteristics consistent with those observed in the TCGA cohort. Single datasets account for 3% (*n* = 5, GSE9891), 8.6% (*n* = 16, GSE26712) and 9% (*n* = 12, GSE32062) of putative CL patients respectively (Figure 7B). Beyond the key genes identifying the CL subtype, a total of 2943 DEGs were identified between CL and all other tumors (non CL tumors), including 1689 up-regulated and 1254 down-regulated genes (1% FDR and >2 or <0.5 fold-change, Figure S5). Of note other genes besides those of the CL minimal signature are altered, including Sodium Channel Epithelial 1 Subunit Alpha (SCNN1A) and Tumor Associated Calcium Signal Transducer 2 (TACSTD), members of cytokeratins family, including KRT7, 8, 18 and 19, and morphogenesis related genes Grainyhead Like Transcription Factor 2 (GRHL2), Kunitz-type protease inhibitor 2 (SPINT2) and Transmembrane Serine Protease Matriptase (ST14).

Gene Set Enrichment Analysis confirmed the enrichment of the EMT pathway and highlights the alteration of other pathways including Kras, DNA damage response and Hedgehog signaling (Figure 7C).

To assess whether the newly identified CL subtype was associated with patient outcomes, we performed survival analysis on overall survival using Cox Proportional Hazard models on individual studies, subsequently summarized using a meta analytic approach. Forest Plot in Figure 7D summarizes the result of the analysis of individual datasets, showing that HGSOC claudin-low are poor outcome tumors compared to others.

All these data support the existence of a CL subtype of HGSOC, which resembles the CL subtype of BC both at the molecular and clinical level, whose molecular profile and morphological characteristics are maintained also when cancer cells are cultured in vitro, further strengthening the translational relevance of this molecular subtype.

## 4. Discussion

Claudin-low is a rare subtype of undifferentiated neoplasms originally described in receptor-negative breast cancers, and more recently in bladder and gastric cancers [22,43], that has been proposed to originate from early epithelial precursors with stemness features [44]. Based on the analysis of publicly available datasets, although they include a limited number of patients, here we provide evidence for the existence of an ovarian HGSOC CL molecular profile, displaying low epithelial differentiation and high mesenchymal signature. CL-HGSOC cell expression profile displays evidence of an EMT pattern, showing inconsistent expression of the cell-cell adhesion cluster containing claudin 3, 4, 7, occludin and E-cadherin, and enrichment in EMT-inducing transcription factors, including direct E-cadherin repressors Snai1 and Snai2, and the master regulator of EMT, Twist1 [45]. Importantly, the CL-HGSOC cell lines well recapitulate the transcriptional characteristics of human ovarian cancers, where the CL tumor cluster is characterized by extremely low expression of genes involved in cell junctional assembly and maintenance such as TACSTD, a paralogous gene of Ep-CAM, together with a massive down regulation of cytokeratins and the morphogenesis related genes GRHL2, SPINT2 and ST14, required to sustain epithelial cell lineage during embryonic development [46]. In epithelial cells Ep-CAM drives intercellular adhesion to ensure tissue integrity by binding claudin in regions distinct from tight junctions [47] and prevents their degradation [48]. In addition, Ep-CAM acts as a negative regulator of cell motility and epithelial cell migration. Thus, loss of Ep-CAM is frequent in tumors during the EMT process, through which epithelial cells lose their morphology and acquire the expression of mesenchymal markers by virtue of actin cytoskeletal rearrangement. EMT allows the acquisition of a high metastatic potential. In addition, EMT may reflect the pliancy of tumors, which can be defined as the intrinsic ability of cancer cells to degenerate upon oncogenic insults. Interestingly, in breast cancer the activation of mitogenic RAS-MAPK pathway mediates EMT and in turn EMT is associated with stemness via the RAS/RAF/MEK/ERK and the RAS/PI3K/AKT pathways [49]. In the regulation of EMT, RAS activity is coordinated by other pathways including p53. In CL breast cancer both p53 and KRAS result altered [44]. Of note, p53 is altered in almost all HGSOC, but rarely co-occurs with KRAS mutation [50]. Among the identified CL-HGSOC cell lines OV7 and OVCAR8 contained both gene alterations, respectively P53^I254S^ and KRAS^G12D^ in OV7, and p53 × ^126splice^ and KRAS ^P121H^ in OVCAR8 (cBioportal https://www.cbioportal.org/ (accessed on 29 October 2020)). In agreement with these observations, GSEA analysis reveals an alteration of p53 and KRAS pathways in CL-HGSOC patients. The presence of mutations in KRAS and the effects of KRAS pathway alteration remain an open point of discussion under investigation in clinical trials of ovarian cancer patients. In colorectal and pancreatic cancer, the presence of mutations in KRAS results in a less responsive tumor when treated with first-generation tyrosine kinase inhibitors like gefitinib. In addition, the presence of mutations precludes patients from treatment with cetuximab and panitumumab, recommended only in the context of wild type KRAS. The effects of KRAS mutation in HGSOC deserve further clinical consideration and investigation.

CL-HGSOC is enriched with stem-like cells in a mesenchymal state, since the majority of cells are CD44^+^/CD24^−^, express mesenchymal markers and display altered response to chemotherapy. Similar stem-like phenotype was observed in CL breast cancers, where stemness is closely related with a high metastatic potential, a resistance to therapy and cancer-related mortality [51]. Thus, we speculate that these features also contribute to the poor outcomes in CL-HGSOC patients. Indeed, CL-HGSOC patients have a worse median overall survival as compared to the others group. These observations suggest potential prognostic utility for clinical practice of this molecular subtyping.

Notably, a mesenchymal/claudin-low population is found at low frequency in OVCAR3 claudin-high model, suggesting that tumors with fully epithelial architecture also possess epithelial cells with mesenchymal properties. In breast cancer, the observed intra-tumor heterogeneity supports the idea that oncogenic events occur in the epithelial mammary stem cells [38], which stop at different differentiation stages [50]. CL tumor derives from the uncommitted stem progenitor. The recent description of a “claudin-lowness” spectrum existing across breast tumors corroborates this model, redefining claudin-low as a continuous feature present at varying degree in individual tumors [52]. Accordingly, a CL subpopulation is present at variable extent in HGSOC cell lines regardless they are classified as CL or “other”. In vitro, the stem-like CL subpopulation isolated from HGSOC labeled as “other” is able to recapitulate the parental cell line in 14 days of culture. Since the origin of ovarian cancer remains elusive, we cannot speculate on the impact of cellular differentiation on the ovarian tumorigenic process.

In contrast to CL bladder cancers [22], currently available data show that CL-HGSOC tumors are not enriched in immune gene signatures associated with specific cellular subpopulations (CD8+ T cells, B cell lineage, Th1-polarizing macrophages) and immunosuppressive gene signatures. Additional studies are required to well characterize the immunological features of these tumors and to evaluate if drugs targeting immune checkpoints could be considered as a therapeutic strategy.

## 5. Conclusions

A claudin-low cancer cell subtype is present in high-grade serous ovarian cancer. Similarly, to other CL cancers, CL-HGSOC displays a signature consistent to mesenchymal and stem cell features. This subtype may represent the most aggressive ovarian cancer associated with a poor prognosis. Future developments of this study will include a multicentric collaboration in order to widen the data collection, as well as the access to larger online datasets, possibly from extended.

The identification of the claudin-low molecular profile may have important clinical implications, paving the way for personalized medicine in ovarian cancer patients.

## Figures and Tables

**Table 1:** TaqMan Gene Expression Assay for target genes obtained from Thermo Fisher as Assay-on-Demand products.

Target	TaqMan Gene Expression Assay ID
claudin-3	Hs00265816-s1
claudin-4	Hs00976831-s1
claudin-7	Hs00600772-m1
occludin	Hs00170162-m1
E-cadherin	Hs01023895-m1
Zeb1	Hs00232783-m1
Snai2	Hs00950344-m1
Twist1	Hs00361186-m1
Twist2	Hs00382379-m1
vimentin	Hs00958111-m1

**Table 2:** Claudin low tumor marker genes.

Gene Symbol	Gene Name	mRNA Expression Levels	References
CLDN3	Claudin 3	down	Prat, A.; et al. [19]; Sabatier, R.; et al. [20]; Perou, C.M. [18].
CLDN4	Claudin 4	down	
CLDN7	Claudin 7	down	
CDH1	E-cadherin	down	
OCLN	Occludin	down	
VIM	Vimentin	up	
SNAI1	Snail-1	up	
SNAI2	Snail-2	up	
TWIST1	Twist1	up	
TWIST2	Twist2	up	
ZEB1	Zinc Finger E-Box Binding Homeobox 1	up	
ZEB2	Zinc Finger E-Box Binding Homeobox 2	up	
CD44	CD44	up	Dias, K.; et al. [21]; Prat, A.; et al. [37].
CD24	CD24	down	
EPCAM (alias TACSTD1)	Epithelial Cell Adhesion Molecule	down	
MUC1	Mucin 1, Cell Surface Associated	down	
ALDH1A1	Aldehyde Dehydrogenase 1 Family Member A1	up	

## Data Availability

The data presented in this study are openly available in [CCLE] at [10.1038/nature11003], reference number [23]; [curatedOvarianData] at [10.1093/database/bat013], reference number [24]; [TCGA] at [10.1038/nature10166], reference number [25]; [Bonome’s dataset] at [10.1158/0008-5472.CAN-07-6595], reference number [26]; [Tothill’s dataset] at [10.1158/1078-0432.CCR-08-0196], reference number [27]; [Japanese dataset] at [10.1158/1078-0432.CCR-11-2725], reference number [28].

## Supplementary Materials

The following are available online at https://www.mdpi.com/2072-6694/13/4/906/s1, Figure S1: Gene set enriched in phenotype claudin-low cell lines. Figure S2: Normalized mRNA levels of indicated genes in MCF-7, OVCAR3, MDA-MB231 and OV7 cell lines measured by qPCR. Figure S3: The original western blotting figures and densitometric analysis. Figure S4: The original western blotting figures sorted CD44^+^/CD24^−^. Figure S5: List of genes differentially expressed (DEGs) between CL and non CL tumors.
