# Supplementary material for: The Claudin-Low Subtype of High-Grade Serous Ovarian Carcinoma Exhibits Stem Cell Features
Source: Cancers (Basel). 2021 Feb 22;13(4):906. doi: 10.3390/cancers13040906 (PMC7926503; doi:10.3390/cancers13040906)
Supplement: Supplementary file 1 [file cancers-13-00906-s001.pdf]

cancers

MDPI

Supplementary Materials

The Claudin-Low Subtype of High-Grade Serous Ovarian Carcinoma Exhibits Stem Cell Features

Chiara Romani, Davide Capoferri, Elisabetta Grillo, Marco Silvestri, Michela Corsini, Laura Zanotti, Paola Todeschini, Antonella Ravaggi, Eliana Bignotti, Franco Odicino, Enrico Sartori, Stefano Calza and Stefania Mitola

NAME	SIZE	ES	NES	NOM p-val	FDR q-val	FWER p-val	RANK AT MAX	LEADING EDGE
HALLMARK_MYC_TARGETS_V1	196	0.67166996	33.764.176	0.0	0.0	0.0	4662	tags=68%, list=22%, signal=87%
HALLMARK_EPITHELIAL_MESENCHYMAL_TRANSITION	197	0.6532058	32.740.583	0.0	0.0	0.0	3216	tags=53%, list=15%, signal=62%
HALLMARK_E2F_TARGETS	198	0.5701431	28.832.994	0.0	0.0	0.0	5023	tags=62%, list=24%, signal=80%
HALLMARK_G2M_CHECKPOINT	195	0.4870398	24.471.917	0.0	0.0	0.0	4712	tags=53%, list=22%, signal=68%
HALLMARK_MYC_TARGETS_V2	57	0.594622	24.343.355	0.0	0.0	0.0	5242	tags=75%, list=25%, signal=100%
HALLMARK_UV_RESPONSE_DN	142	0.4655522	22.682.207	0.0	0.0	0.0	2724	tags=35%, list=13%, signal=40%
HALLMARK_REACTIVE_OXYGEN_SPECIES_PATHWAY	49	0.52316475	2.075.629	0.0	0.0	0.0	3341	tags=37%, list=16%, signal=43%
HALLMARK_HYPOXIA	195	0.39383224	19.568.353	0.0	2,14E+03	0.001	3297	tags=33%, list=16%, signal=39%
HALLMARK_ANDROGEN_RESPONSE	97	0.4127399	18.873.345	0.0	4,76E+02	0.002	2452	tags=31%, list=12%, signal=35%
HALLMARK_DNA_REPAIR	146	0.38372913	18.547.115	0.0	4,29E+03	0.002	4948	tags=50%, list=23%, signal=65%
HALLMARK_MTORC1_SIGNALING	197	0.35252362	18.007.857	0.0	0.001185124	0.006	3359	tags=31%, list=16%, signal=36%
HALLMARK_TNFA_SIGNALING_VIA_NFKB	194	0.3575453	17.980.323	0.0	0.0010863637	0.006	2868	tags=28%, list=13%, signal=32%
HALLMARK_UNFOLDED_PROTEIN_RESPONSE	111	0.3863376	17.884.074	0.0	0.0010027973	0.006	4236	tags=41%, list=20%, signal=50%
HALLMARK_KRAS_SIGNALING_UP	196	0.35548317	17.773.813	0.0	9,31E+02	0.006	2699	tags=26%, list=13%, signal=29%
HALLMARK_MITOTIC_SPINDLE	196	0.34008372	17.374.634	0.0	0.0017647771	0.012	3261	tags=32%, list=15%, signal=37%
HALLMARK_COMPLEMENT	195	0.3384013	17.041.323	0.0	0.002611149	0.019	2525	tags=22%, list=12%, signal=25%
HALLMARK_APICAL_JUNCTION	198	0.32970953	16.841.704	0.0	0.0032836024	0.025	3512	tags=32%, list=17%, signal=38%
HALLMARK_INFLAMMATORY_RESPONSE	199	0.32807446	16.580.292	0.0	0.003970451	0.032	2819	tags=24%, list=13%, signal=28%
HALLMARK_APOPTOSIS	160	0.31447122	153.043	0.003076923	0.012039007	0.107	3251	tags=29%, list=15%, signal=34%
HALLMARK_GLYCOLYSIS	195	0.28923222	1.460.143	0.003125	0.023048187	0.204	3243	tags=26%, list=15%, signal=31%
HALLMARK_TGF_BETA_SIGNALING	54	0.36065137	14.508.951	0.032098766	0.024409335	0.223	2110	tags=28%, list=10%, signal=31%
HALLMARK_COAGULATION	135	0.29133725	1.393.121	0.017291067	0.038923804	0.345	2410	tags=21%, list=11%, signal=24%
HALLMARK_PROTEIN_SECRETION	95	0.3058714	13.818.902	0.04034582	0.04254744	0.386	4314	tags=37%, list=20%, signal=46%
HALLMARK_IL2_STAT5_SIGNALING	198	0.26350182	13.250.142	0.015723271	0.066279806	0.541	3485	tags=27%, list=16%, signal=32%
HALLMARK_ALLOGRAFT_REJECTION	197	0.2588365	13.007.649	0.032894738	0.077414535	0.61	2782	tags=19%, list=13%, signal=21%
HALLMARK_MYOGENESIS	196	0.25334948	12.943.505	0.018404908	0.07909152	0.63	2585	tags=19%, list=12%, signal=21%
HALLMARK_SPERMATOGENESIS	134	0.26374075	1.262.039	0.06388889	0.0978708	0.718	3387	tags=25%, list=16%, signal=30%
HALLMARK_XENOBIOTIC_METABOLISM	199	0.24794042	12.447.128	0.06344411	0.10786284	0.757	2027	tags=15%, list=10%, signal=17%
HALLMARK_WNT_BETA_CATENIN_SIGNALING	42	0.31839064	12.107.011	0.15068494	0.13499121	0.847	1965	tags=19%, list=9%, signal=21%
HALLMARK_PI3K_AKT_MTOR_SIGNALING	104	0.23627277	10.960.205	0.2820513	0.29831013	0.988	3558	tags=27%, list=17%, signal=32%
HALLMARK_OXIDATIVE_PHOSPHORYLATION	198	0.21179302	10.771.439	0.24522293	0.32989255	0.99	4428	tags=30%, list=21%, signal=37%
HALLMARK_IL6_JAK_STAT3_SIGNALING	86	0.24117215	10.631.292	0.3042328	0.3490553	0.992	2312	tags=17%, list=11%, signal=19%
HALLMARK_FATTY_ACID_METABOLISM	156	0.21785496	10.572.335	0.31707317	0.35163328	0.994	2856	tags=19%, list=13%, signal=21%
HALLMARK_ADIPOGENESIS	199	0.2078379	10.468.153	0.29139072	0.36284268	0.996	4304	tags=31%, list=20%, signal=39%
HALLMARK_ANGIOGENESIS	36	0.28207684	10.390.545	0.4059406	0.37094843	0.996	2396	tags=19%, list=11%, signal=22%
HALLMARK_PANCREAS_BETA_CELLS	40	0.22175238	0.85355693	0.68041235	0.82055134	1.0	2708	tags=18%, list=13%, signal=20%

Figure S1. Gene set enriched in phenotype claudin-low.

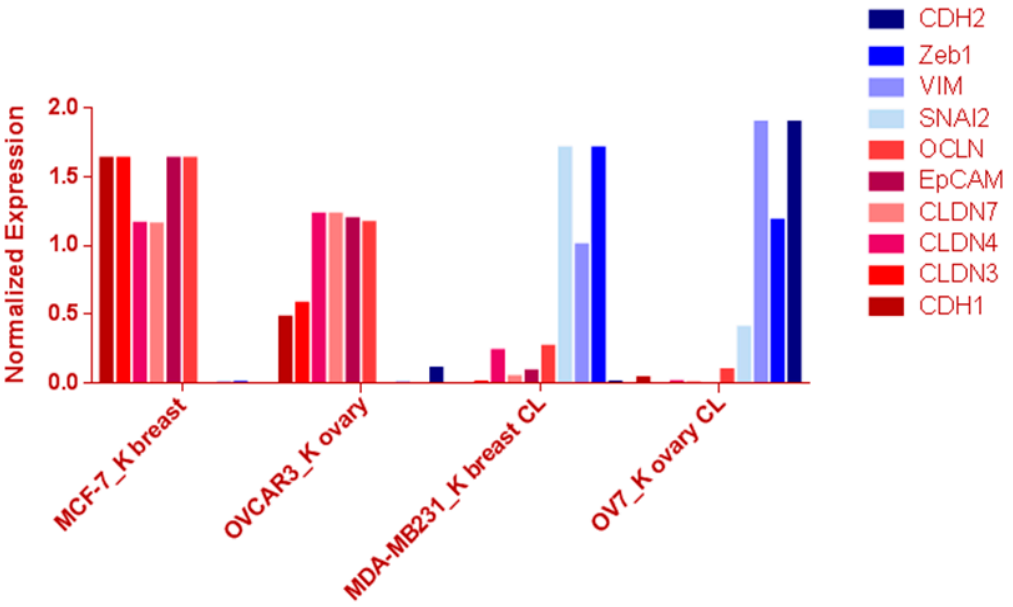

Figure S2. Normalized mRNA levels of indicated genes in MCF-7, OVCAR3, MDA-MB231 and OV7 cell lines measured by qPCR.

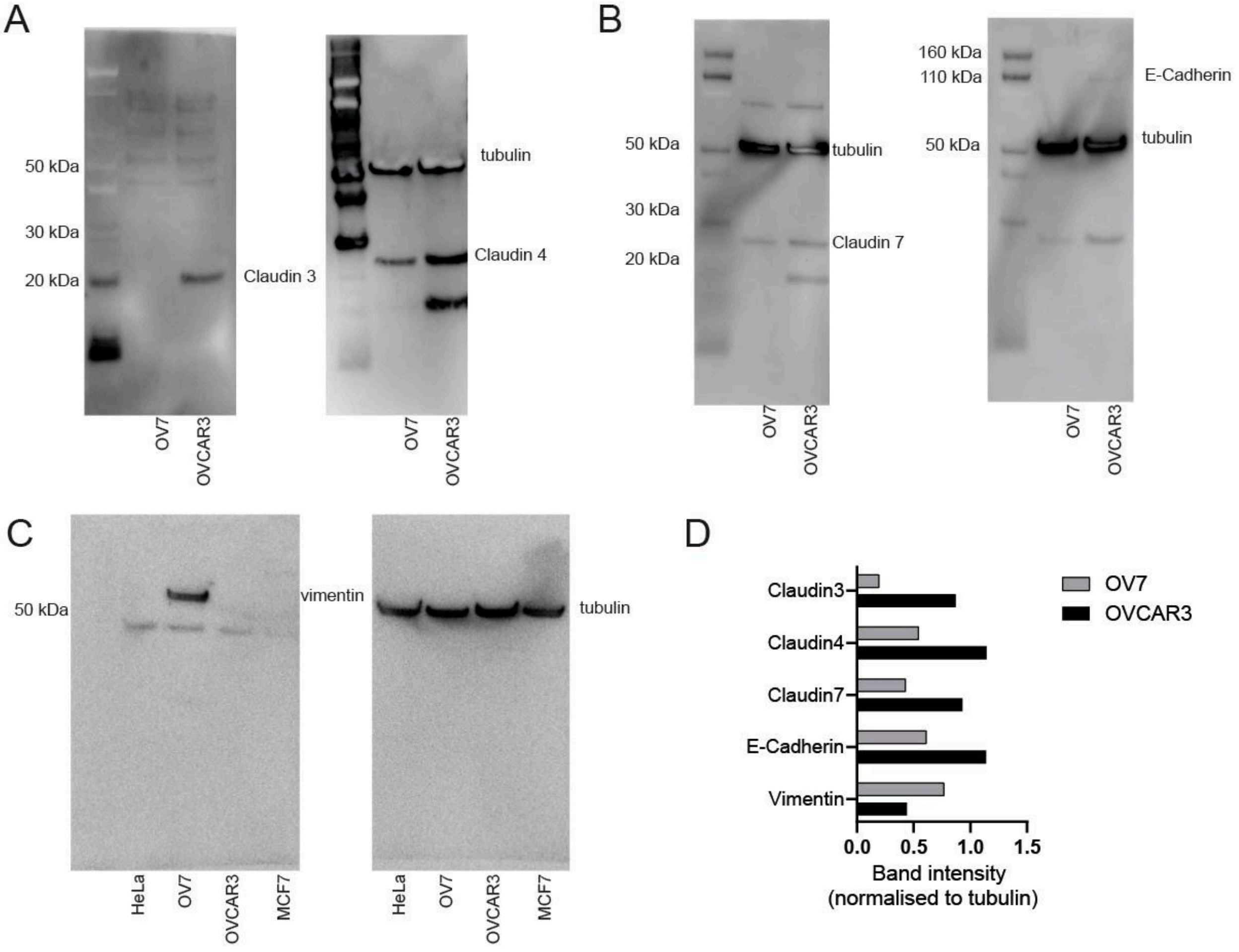

Figure S3. (**A-C**), full Western blot membrane shown as cropped images in Main Figure 2. Specific bands and molecular weights are indicated. (**D**), levels of protein expression in OV7 and OVCAR3 cell lines were measured by Western blot densitometry and are shown as band intensity normalized to tubulin levels.

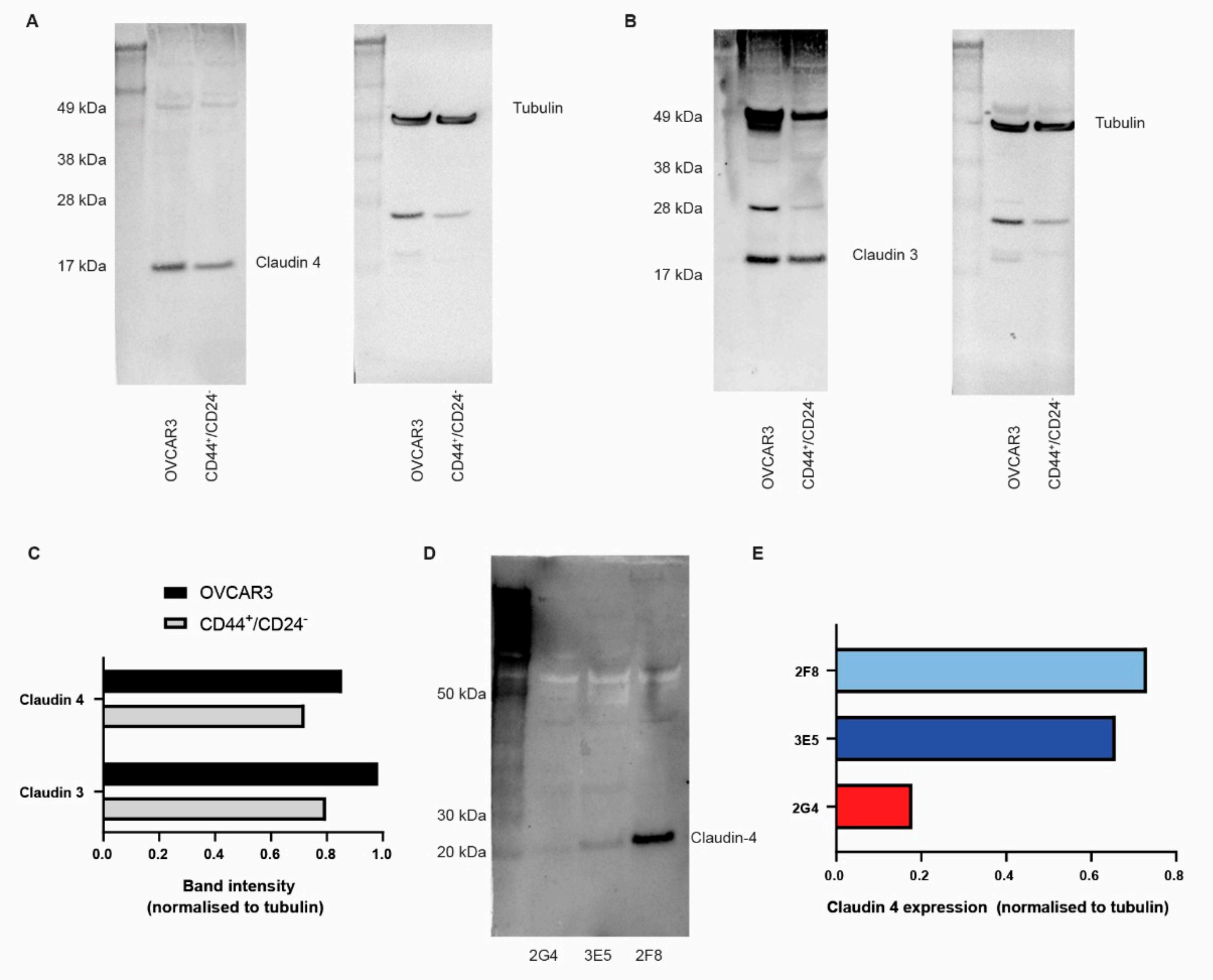

Figure S4. (**A**,**B**), western blot analysis of claudins expression in unsorted OVCAR3 and CD44+/CD24- sorted OVCAR3; (C) densitometric analysis of WB. (**D**), Claudin 4 expression in 3 OVCAR 3 clone s-.

HUGO	FDR	FC (CL vs non-CL)	FDR
SYDE1	5,43038E-19	3,023	< 0.01
CPEB1	4,49265E-32	2,854	< 0.01
NUDT11	1,36825E-28	2,770	< 0.01
SEPT6	4,38E-17	2,714	< 0.01
PENK	1,34727E-42	2,686	< 0.01
ANGPT1	1,40664E-18	2,684	< 0.01
CDON	1,03156E-17	2,632	< 0.01
PPT2	1,48313E-17	2,626	< 0.01
ATP1B2	1,3096E-24	2,592	< 0.01
NOVA1	4,22606E-16	2,563	< 0.01
KCTD20	8,77253E-17	2,559	< 0.01
TUBB4A	5,72706E-20	2,544	< 0.01
MAGEL2	7,48815E-24	2,464	< 0.01
C2orf44	1,05364E-14	2,451	< 0.01
JAKMIP2	1,72062E-20	2,426	< 0.01
ATP8B2	3,1634E-14	2,411	< 0.01
CSRP3	1,05364E-14	2,400	< 0.01
TSPYL2	5,55222E-13	2,387	< 0.01
C3orf18	2,84905E-12	2,377	< 0.01
FGF2	5,42573E-18	2,364	< 0.01
SALL2	7,26501E-13	2,360	< 0.01
CCDC88A	1,21795E-15	2,341	< 0.01
GJC1	1,11962E-13	2,340	< 0.01
ZC4H2	7,87502E-13	2,307	< 0.01
B3GNT1	1,63209E-12	2,304	< 0.01
STARD8	7,47084E-12	2,301	< 0.01
DENND2A	6,8722E-14	2,298	< 0.01
FNDC4	2,79169E-13	2,296	< 0.01
CXXC4	1,66213E-15	2,292	< 0.01
FSD1	1,61529E-16	2,288	< 0.01
SV2A	1,12797E-12	2,287	< 0.01
KCTD7	3,03855E-11	2,268	< 0.01
IL11RA	8,00804E-11	2,263	< 0.01
AGPAT4	4,18633E-13	2,260	< 0.01
MRAS	2,20889E-12	2,252	< 0.01
PRR3	6,5327E-12	2,240	< 0.01
DGKD	7,24846E-12	2,238	< 0.01
CORO2B	6,05476E-16	2,237	< 0.01
ARHGEF40	1,91159E-12	2,234	< 0.01
PTPRD	7,89895E-13	2,226	< 0.01
PRKAB2	7,43662E-13	2,219	< 0.01
TBKBP1	6,1547E-11	2,218	< 0.01
JAM2	1,38943E-11	2,217	< 0.01
DZIP1	2,86231E-12	2,217	< 0.01
PPM1F	4,66736E-13	2,210	< 0.01
CNTLN	7,40432E-14	2,204	< 0.01
SNCA	3,02368E-11	2,193	< 0.01
PYGO1	4,0429E-13	2,182	< 0.01
DDX25	3,18391E-21	2,181	< 0.01
HSPB6	1,17437E-15	2,173	< 0.01
CHST10	4,39344E-10	2,169	< 0.01
PTH1R	1,29246E-14	2,168	< 0.01
TTBK2	1,97024E-13	2,160	< 0.01
MAPK10	2,01058E-15	2,158	< 0.01
GOLIM4	5,71939E-13	2,157	< 0.01
VASH2	5,88206E-12	2,146	< 0.01
EFS	2,15224E-09	2,146	< 0.01
CSPG4	1,0017E-12	2,144	< 0.01

CCIN	5,82587E-13	2,134	< 0.01
BEND5	2,8832E-09	2,130	< 0.01
MPDZ	1,16144E-11	2,130	< 0.01
GREB1L	1,9264E-11	2,128	< 0.01
BEGAIN	5,69067E-11	2,128	< 0.01
DCHS1	4,71946E-09	2,128	< 0.01
CD22	1,19021E-12	2,114	< 0.01
LSAMP	4,13291E-13	2,106	< 0.01
SEMA6A	3,62954E-12	2,101	< 0.01
FGFR1	6,69255E-10	2,092	< 0.01
ZCCHC24	1,71501E-09	2,090	< 0.01
MAP1A	7,659E-12	2,090	< 0.01
FLNC	5,13229E-13	2,089	< 0.01
HRC	3,57694E-14	2,088	< 0.01
GLI2	6,21575E-10	2,088	< 0.01
EXOG	1,00282E-09	2,080	< 0.01
GNAZ	7,45456E-10	2,080	< 0.01
GPR176	1,1139E-09	2,078	< 0.01
GATA2	1,42632E-14	2,077	< 0.01
RHOBTB3	4,62327E-09	2,071	< 0.01
FHL3	1,3061E-10	2,070	< 0.01
AASS	1,31597E-10	2,069	< 0.01
CXorf57	1,912E-19	2,064	< 0.01
QKI	6,80683E-10	2,060	< 0.01
LMBR1L	2,03772E-09	2,059	< 0.01
AMHR2	4,02915E-10	2,054	< 0.01
NRXN2	3,17594E-13	2,052	< 0.01
AXL	1,5859E-09	2,050	< 0.01
CDC5L	7,47186E-11	2,045	< 0.01
LEFTY2	9,29845E-15	2,043	< 0.01
SLC35G2	1,66351E-11	2,043	< 0.01
NLRP1	1,28197E-10	2,033	< 0.01
CAP2	1,47582E-10	2,032	< 0.01
MAP3K3	3,54937E-09	2,022	< 0.01
SNCAIP	1,19374E-09	2,020	< 0.01
PKIA	1,25915E-08	2,018	< 0.01
CREB5	6,6162E-12	2,017	< 0.01
MYOZ3	1,25793E-09	2,016	< 0.01
TJAP1	2,95801E-10	2,013	< 0.01
GNG4	1,13615E-16	2,013	< 0.01
ACTN2	8,20067E-15	2,010	< 0.01
CACNB4	6,80622E-12	2,010	< 0.01
CACNA2D1	6,75872E-09	2,009	< 0.01
NAALAD2	1,2901E-17	2,008	< 0.01
CAMK2A	4,26773E-09	2,006	< 0.01
KLHL20	7,69842E-09	2,001	< 0.01
TNFRSF21	1,91148E-09	0,500	< 0.01
PTGS1	9,15955E-10	0,499	< 0.01
ATP5H	1,29762E-09	0,499	< 0.01
MTIF2	8,59557E-10	0,499	< 0.01
CELSR1	3,84593E-09	0,498	< 0.01
S100A13	4,94922E-10	0,498	< 0.01
FKBP2	4,54682E-09	0,498	< 0.01
CD2AP	1,37901E-08	0,497	< 0.01
SIGIRR	2,46543E-09	0,497	< 0.01
MTX2	7,55578E-09	0,496	< 0.01
HSPB1	2,64099E-09	0,495	< 0.01
SLC44A4	5,96174E-09	0,495	< 0.01
LRP10	1,03153E-09	0,494	< 0.01

HNRNPH2	2,39873E-09	0,494	< 0.01
DERL1	2,00158E-08	0,494	< 0.01
PDCD6	9,27324E-09	0,494	< 0.01
PPFIBP2	6,09031E-10	0,494	< 0.01
RNASET2	7,51066E-09	0,494	< 0.01
IRAK1	4,32644E-10	0,494	< 0.01
LYPD1	1,56418E-08	0,494	< 0.01
DERA	3,90274E-10	0,493	< 0.01
SRPR	1,41016E-09	0,493	< 0.01
COPS7A	2,33448E-09	0,493	< 0.01
NDUFA1	2,05063E-09	0,492	< 0.01
PITRM1	4,19254E-09	0,492	< 0.01
GFPT1	4,62186E-09	0,492	< 0.01
CTBP2P4	1,01517E-10	0,492	< 0.01
C9orf16	2,57586E-10	0,492	< 0.01
PRR15L	1,54227E-08	0,492	< 0.01
TEX264	1,12679E-07	0,491	< 0.01
AHCY	8,0899E-09	0,491	< 0.01
ORMDL2	2,58626E-09	0,490	< 0.01
SORD	3,21909E-09	0,489	< 0.01
PSME2	3,71359E-09	0,489	< 0.01
BSPRY	8,45038E-10	0,489	< 0.01
FAM173A	1,30974E-08	0,488	< 0.01
PRKX	4,65464E-10	0,488	< 0.01
SLC34A2	1,74825E-10	0,487	< 0.01
C3	2,78886E-11	0,487	< 0.01
AIM1	3,43403E-10	0,487	< 0.01
ATP6AP2	6,09031E-10	0,486	< 0.01
EIF2AK1	1,40423E-08	0,486	< 0.01
PLEKHB2	6,17013E-11	0,485	< 0.01
HN1L	2,62711E-08	0,485	< 0.01
GHITM	9,91294E-11	0,485	< 0.01
GPI	2,94105E-10	0,484	< 0.01
GPX1	1,21161E-10	0,484	< 0.01
TM9SF1	4,81074E-10	0,484	< 0.01
ANXA2	4,69087E-10	0,483	< 0.01
COX8A	1,41016E-09	0,483	< 0.01
HLA-DRB5	3,37817E-10	0,483	< 0.01
AKT1	8,84064E-10	0,482	< 0.01
SLC25A5	2,38663E-10	0,482	< 0.01
EFR3A	1,22867E-10	0,482	< 0.01
ANXA2P2	4,92942E-10	0,482	< 0.01
FLII	1,35011E-10	0,481	< 0.01
NDUFV2P1	3,38356E-09	0,481	< 0.01
NDUFV2	3,38356E-09	0,481	< 0.01
MLF2	1,28852E-10	0,481	< 0.01
TSPAN12	6,5673E-09	0,480	< 0.01
RHBDF2	3,67909E-10	0,480	< 0.01
FZD6	8,61578E-10	0,479	< 0.01
JUP	1,37938E-11	0,479	< 0.01
SLC38A1	5,85545E-12	0,478	< 0.01
ALDH3B2	9,94032E-09	0,477	< 0.01
CDS1	8,7405E-10	0,476	< 0.01
NMI	1,99597E-10	0,476	< 0.01
TNFRSF1A	8,79512E-10	0,476	< 0.01
PFN1	9,97069E-13	0,476	< 0.01
MRPL12	4,80997E-09	0,475	< 0.01
ZNF706	1,29806E-09	0,475	< 0.01
IFI27	6,80622E-12	0,475	< 0.01

IRF6	2,15852E-09	0,475	< 0.01
PPIF	2,29965E-10	0,471	< 0.01
PHLDA2	3,62158E-09	0,471	< 0.01
TAGLN2P1	2,06788E-11	0,471	< 0.01
YIF1A	3,77464E-10	0,471	< 0.01
NADSYN1	4,47071E-09	0,471	< 0.01
KIAA0247	2,30582E-09	0,470	< 0.01
SLC22A18	1,29762E-09	0,470	< 0.01
KLK11	3,39604E-10	0,469	< 0.01
CCDC47	1,62975E-09	0,469	< 0.01
ZDHHC13	3,94698E-10	0,469	< 0.01
CHMP3	1,90214E-12	0,469	< 0.01
DSG2	5,01537E-12	0,467	< 0.01
ALDOA	4,24881E-10	0,467	< 0.01
RTN3	1,31331E-10	0,465	< 0.01
RALBP1	2,82657E-10	0,465	< 0.01
TAPBPL	3,40812E-09	0,464	< 0.01
CMTM6	3,34202E-10	0,463	< 0.01
ACO2	3,22895E-12	0,462	< 0.01
SMPDL3B	5,33881E-11	0,459	< 0.01
TMX2	7,01742E-12	0,457	< 0.01
CSTB	4,13234E-12	0,457	< 0.01
ATP6V0C	1,2198E-10	0,457	< 0.01
KLK6	2,58542E-12	0,456	< 0.01
PSME1	6,07927E-10	0,456	< 0.01
ITPR3	1,62592E-10	0,455	< 0.01
MPZL2	1,4369E-10	0,454	< 0.01
KLK9	7,80333E-11	0,454	< 0.01
GALNT6	5,28945E-11	0,454	< 0.01
WDR45B	7,56108E-11	0,453	< 0.01
RGS10	2,25129E-11	0,452	< 0.01
ATP6AP1	6,82459E-13	0,452	< 0.01
TJP3	1,66603E-10	0,451	< 0.01
PDZK1IP1	1,17852E-10	0,451	< 0.01
TMBIM6	9,15188E-12	0,450	< 0.01
FAM120A	2,31116E-14	0,450	< 0.01
SCP2	3,1634E-14	0,450	< 0.01
MYO6	1,3538E-11	0,450	< 0.01
PRKCI	6,93658E-11	0,450	< 0.01
CNDP2	1,697E-11	0,450	< 0.01
LRBA	3,44574E-11	0,449	< 0.01
LAD1	2,5243E-14	0,447	< 0.01
PPP1CA	1,14126E-12	0,446	< 0.01
SOX17	4,33199E-18	0,446	< 0.01
ZBED2	7,08925E-10	0,446	< 0.01
LTBR	3,56645E-14	0,445	< 0.01
IQGAP1	4,90796E-12	0,445	< 0.01
NARS	1,26307E-11	0,445	< 0.01
BCL2L1	1,3538E-11	0,445	< 0.01
TPI1	1,22715E-12	0,444	< 0.01
PKM	8,82871E-13	0,443	< 0.01
CXADR	9,12519E-17	0,443	< 0.01
EHF	3,70055E-12	0,443	< 0.01
ESRP2	5,33881E-11	0,442	< 0.01
TWF1	2,0106E-12	0,442	< 0.01
WWC1	2,61467E-11	0,442	< 0.01
PPP1R14B	2,43476E-12	0,441	< 0.01
POR	5,42355E-13	0,440	< 0.01
AIFM1	3,7656E-11	0,439	< 0.01

ARPC1B	4,81918E-13	0,438	< 0.01
ELMO3	1,22469E-11	0,437	< 0.01
SLC35A2	2,95279E-14	0,437	< 0.01
BHLHE41	7,33139E-18	0,437	< 0.01
WBSCR22	1,56574E-12	0,436	< 0.01
GALNT3	6,56887E-12	0,436	< 0.01
IDH2	6,78975E-13	0,435	< 0.01
COX5A	4,18607E-13	0,434	< 0.01
MMP7	1,3061E-10	0,434	< 0.01
S100A11	3,0556E-16	0,432	< 0.01
VTCN1	4,22761E-12	0,430	< 0.01
CFB	3,8542E-13	0,429	< 0.01
LAMC2	1,68387E-12	0,428	< 0.01
KLF5	1,4116E-13	0,428	< 0.01
PRKCD	2,55248E-12	0,426	< 0.01
RIPK4	3,28485E-13	0,424	< 0.01
TAPBP	2,93223E-13	0,424	< 0.01
PFKP	5,51071E-16	0,424	< 0.01
FRAT2	1,25751E-12	0,424	< 0.01
LAPTM4B	5,95002E-14	0,423	< 0.01
TPI1P1	4,38601E-15	0,422	< 0.01
PPA1	2,15816E-15	0,422	< 0.01
LGALS3	5,28248E-14	0,422	< 0.01
PPP1R14BP3	8,20208E-14	0,422	< 0.01
DPP3	2,19914E-12	0,422	< 0.01
CD47	3,11452E-16	0,421	< 0.01
ITGB4	3,82915E-12	0,420	< 0.01
F2RL1	1,76094E-11	0,419	< 0.01
MGAT4B	3,30871E-14	0,415	< 0.01
TFAP2C	1,60652E-14	0,414	< 0.01
BACE2	2,49687E-15	0,413	< 0.01
CP	2,51672E-18	0,411	< 0.01
FOLR1	7,17713E-18	0,410	< 0.01
ZNF217	1,45097E-14	0,409	< 0.01
GSTK1	1,18953E-14	0,409	< 0.01
PCBD1	1,57052E-14	0,408	< 0.01
CD74	8,47366E-17	0,406	< 0.01
SDC4	1,50911E-13	0,405	< 0.01
CRIP1	2,29331E-16	0,403	< 0.01
LGALS3BP	6,042E-16	0,400	< 0.01
S100A14	4,53656E-14	0,399	< 0.01
KLK8	2,65342E-17	0,399	< 0.01
PTPRF	1,04155E-16	0,398	< 0.01
MGST2	4,28269E-17	0,397	< 0.01
KLK10	7,33553E-15	0,396	< 0.01
LAMB3	4,56718E-13	0,396	< 0.01
RAB25	6,27994E-14	0,395	< 0.01
MBD2	1,39898E-15	0,392	< 0.01
GSTP1	2,12772E-18	0,390	< 0.01
GRHL2	6,56842E-14	0,389	< 0.01
BIK	1,44309E-16	0,389	< 0.01
EPS8L2	1,091E-14	0,388	< 0.01
FXYD3	1,29246E-14	0,387	< 0.01
PTPN6	1,21719E-15	0,387	< 0.01
ANXA1	2,70634E-18	0,387	< 0.01
ASS1	5,78638E-19	0,385	< 0.01
PPL	2,60008E-15	0,385	< 0.01
TPD52	5,78326E-17	0,384	< 0.01
KLK7	4,62592E-20	0,383	< 0.01

SH3YL1	6,20562E-16	0,379	< 0.01
C1orf106	7,33139E-18	0,376	< 0.01
CAPN1	4,35485E-18	0,375	< 0.01
SGPL1	5,27188E-18	0,375	< 0.01
MYOF	1,14981E-18	0,375	< 0.01
CDCP1	8,53768E-16	0,375	< 0.01
ESRP1	6,99068E-19	0,374	< 0.01
EPHA1	5,18371E-19	0,373	< 0.01
MSLN	1,082E-20	0,370	< 0.01
SPON1	1,52321E-30	0,370	< 0.01
GPR56	8,6585E-17	0,370	< 0.01
DHCR24	1,56675E-18	0,368	< 0.01
PKP3	1,71816E-18	0,363	< 0.01
WFDC2	1,8407E-26	0,359	< 0.01
TJP2	4,35485E-18	0,356	< 0.01
F11R	3,8852E-19	0,343	< 0.01
CYBA	3,42646E-21	0,341	< 0.01
ARHGAP8	1,0825E-18	0,341	< 0.01
DENND2D	5,94608E-23	0,337	< 0.01
LLGL2	1,14981E-18	0,336	< 0.01
EPS8L1	1,86117E-20	0,336	< 0.01
UCP2	4,38721E-22	0,336	< 0.01
MUC1	3,66988E-23	0,329	< 0.01
ANXA11	3,17562E-24	0,326	< 0.01
PERP	2,21205E-23	0,326	< 0.01
KRT19	3,13927E-25	0,326	< 0.01
TMPRSS3	9,31373E-22	0,324	< 0.01
KRT18	1,3096E-24	0,321	< 0.01
PRR5-ARHGAP8	6,63343E-20	0,320	< 0.01
ELF3	7,74732E-27	0,315	< 0.01
ST14	3,84822E-24	0,313	< 0.01
SYNGR2	2,73771E-25	0,311	< 0.01
AP1M2	4,39109E-22	0,309	< 0.01
ERBB3	3,13927E-25	0,308	< 0.01
KRT8P3	2,60786E-25	0,305	< 0.01
SLPI	9,75439E-31	0,304	< 0.01
KRT8	7,24979E-26	0,303	< 0.01
CD9	3,18803E-28	0,301	< 0.01
ABHD11	3,46523E-25	0,300	< 0.01
OVOL2	2,31724E-28	0,300	< 0.01
TMEM30B	1,80261E-27	0,296	< 0.01
LSR	1,69475E-27	0,288	< 0.01
DSP	2,94226E-34	0,281	< 0.01
SPINT2	8,87004E-32	0,273	< 0.01
MUC16	1,19184E-35	0,272	< 0.01
PRSS8	8,35619E-36	0,261	< 0.01
VAMP8	2,45856E-38	0,253	< 0.01
TACSTD2	2,04995E-38	0,241	< 0.01
RBM47	1,38608E-37	0,241	< 0.01
KRT7	7,26563E-41	0,232	< 0.01
SPINT1	5,91095E-42	0,223	< 0.01
EPCAM	6,11512E-78	0,193	< 0.01
SCNN1A	4,56558E-71	0,170	< 0.01

Figure S5. List of genes differentially expressed (DEGs) between CL and non CL tumors.
